# Temperature and microbe mediated impacts of the San Diego Bay ostreid herpesvirus (OsHV-1) microvariant on juvenile Pacific oysters

**DOI:** 10.1093/sumbio/qvae014

**Published:** 2024-06-22

**Authors:** Emily Kunselman, Daysi Manrique, Colleen A Burge, Sarah Allard, Zachary Daniel, Guillaume Mitta, Bruno Petton, Jack A Gilbert

**Affiliations:** University of California, San Diego, Scripps Institution of Oceanography, Center for Marine Biotechnology and Biomedicine, 8750 Biological Grade, La Jolla, CA 92037, United States; Pomona College, Biology Department, 333 N College Way, Claremont, CA 91711, United States; California Department of Fish and Wildlife, UC Davis Bodega Marine Laboratory, 2099 Westshore Road, Bodega Bay, CA 94923, United States; University of California, San Diego, Scripps Institution of Oceanography, Center for Marine Biotechnology and Biomedicine, 8750 Biological Grade, La Jolla, CA 92037, United States; University of California, San Diego, Department of Pediatrics, 9500 Gilman Dr, La Jolla, CA 92093, United States; University of California, San Diego, Scripps Institution of Oceanography, Center for Marine Biotechnology and Biomedicine, 8750 Biological Grade, La Jolla, CA 92037, United States; Ifremer, ILM, IRD, UPF, UMR 241 SECOPOL, BP 49 - 98725 Vairao - Tahiti, French Polynesia; Univ Brest, Ifremer, CNRS, IRD, LEMAR, F-29280 Plouzané, France; University of California, San Diego, Scripps Institution of Oceanography, Center for Marine Biotechnology and Biomedicine, 8750 Biological Grade, La Jolla, CA 92037, United States; University of California, San Diego, Department of Pediatrics, 9500 Gilman Dr, La Jolla, CA 92093, United States

**Keywords:** applied microbiology, aquaculture, climate, diseases, microbial ecology, microbiome, shellfish, virus

## Abstract

The ostreid herpesvirus (OsHV-1) was recently detected in San Diego Bay for the first time in farmed juvenile Pacific oysters (*Crassostrea gigas*). Due to the virus’ ability to cause mass mortality (50%–100%), it is important to determine the factors that promote infection as well as the consequences of infection. Here, we assess the role of temperature in controlling OsHV-1 induced mortality. Pacific oysters were exposed to the San Diego Bay microvariant of OsHV-1 at four different temperatures (15°C, 18°C, 21°C, and 24°C). While OsHV-1 was able to replicate in oyster tissues at all temperatures, it did not induce mortality at 15°C, only at the higher temperatures. Additionally, we examined oyster tissue-associated bacterial response to OsHV-1 infection. As shown previously, bacterial richness increased following OsHV-1 exposure and then decreased as the oysters became sick and died. Four bacterial taxa linked to the San Diego Bay microvariant infection, including *Arcobacter, Vibrio, Amphritea*, and *Pseudoalteromonas*, were the same as those shown for other microvariant infections in other studies from globally distributed oysters, suggesting a similar spectrum of co-infection irrespective of geography and microvariant type. The significant shift in the bacterial community following exposure suggests a weakening of the host defenses as a result of OsHV-1 infection, which potentially leads to adverse opportunistic bacterial infection.

**Sustainability Statement**

The temperature threshold of OsHV-1 microvariant from San Diego Bay is critical information for management of the virus. With global ocean temperatures rising, the likelihood of OsHV-1-induced mortality outbreaks may increase. Microbial sequencing also further elucidated the presence of genus-specific bacterial activity in association with OsHV-1, which may be critical in disease progression and potential targets for combatting oyster disease. The impacts that OsHV-1 has on oyster aquaculture affect both food security (UN Sustainable Development Goal 2) and ocean sustainability (UN Sustainable Development Goal 14) because growth of aquaculture is essential to addressing seafood demand but disease outbreaks can cause negative consequences to both food supply and the health of the environment around those outbreaks.

## Introduction

The ostreid herpesvirus (OsHV-1) is a pathogen that plagues aquaculture industries around the world, namely in France, Italy, Ireland, New Zealand, Australia, and the West Coast of the United States (Burge et al. 2006, 2021, Segarra et al. 2010, Lynch et al. 2012, Martenot et al. 2012, Peeler et al. 2012, Jenkins et al. 2013, Keeling et al. 2014, Burioli et al. 2016, 2017, Abbadi et al. 2018). Microvariants of OsHV-1 (µVar), which carry sequence variations from the reference genome (Davison et al. 2005), have evolved. They are highly virulent and lead to mass mortality events on oyster farms (Jenkins et al. 2013, Keeling et al. 2014, Burge et al. 2021, Mazaleyrat et al. 2022). Herpesvirus particles are seen in microscopy across various tissues, including adductor muscle, mantle and digestive gland, and specifically target oyster immune cells called hemocytes, which may act as vehicles transporting the viral particles to different tissues (Schikorski et al. 2011, Martenot et al. 2017, de Lorgeril et al. 2018b). The virus attaches to host cells to invade them and replicate within (Martenot et al. 2019). Inhibition of apoptosis is frequently found in susceptible oysters and may be caused by viral reprogramming in favor of its own proliferation (Green et al. 2016, Martenot et al. 2017, de Lorgeril et al. 2018b, Picot et al. 2022). OsHV-1 may be detected and quantified by both DNA copies and mRNA expression levels, with quantitative PCR (polymerase chain reaction) (Martenot et al. 2010, Burge and Friedman 2012, Agnew et al. 2020, Burge et al. 2020, 2021). Controlled laboratory studies have shown that OsHV-1 may be transmitted by filtered viral homogenate into naïve oysters through intramuscular injection, bath exposure with tissue homogenates, or bath exposure using shed virus (Schikorski et al. 2011, de Lorgeril et al. 2018b, Burge et al. 2020). The virus is also spread through cohabitation, suggesting inter-oyster transmission on farms and in natural populations during outbreaks (Petton et al. 2013, de Lorgeril et al. 2018b).

OsHV-1 virulence is exacerbated by increasing temperatures. In fact, certain microvariants of OsHV-1 are incapable of inducing mortality below certain temperatures. An Australian microvariant was only found to induce mortality above 14°C (de Kantzow et al. 2016). Other microvariants, such as the French microvariant, have greatly reduced replication and almost no oyster mortality at lower temperatures such as 13°C (Pernet et al. 2015). As temperatures are increased, OsHV-1 induced mortality can increase up to 57% (μVar, Delisle et al. 2018), 84% (μVar, de Kantzow et al. 2016), 89.8% (μVar, Petton et al. 2013), or even 100% (Burge et al. 2007). The most optimal range for most OsHV-1 microvariants appears to be between 16°C and 26°C (Burge et al. 2007, Petton et al. 2013, Pernet et al. 2015, de Kantzow et al. 2016, Delisle et al. 2018, Pathirana et al. 2022). Optimal growth and function temperature for Pacific oysters is typically around 20°C (Wiltshire 2007). Higher temperatures may interfere with antiviral gene expression and cause differential response to OsHV-1, leading to higher likelihood of mortality (Green et al. 2014).

In San Diego Bay, temperatures vary from 15°C in winter to more than 22°C in summer (NOAA National Data Buoy Center: https://www.ndbc.noaa.gov/view_climplot.php?station=sdbc1&meas=st). Temperatures reach as high as 25°C in San Diego Bay. The first detection of an OsHV-1 microvariant in San Diego Bay occurred in 2018 in farmed triploid juvenile Pacific oysters (*Crassostrea gigas*) (Burge et al. 2021). The detection of high OsHV-1 copy numbers in October of 2018 coincided with 99% spat mortality (Burge et al. 2021). This is a microvariant of OsHV-1, which is highly virulent, similar to French and Australian microvariants (Kachmar et al. in press). There have since been ongoing sentinel studies to monitor the spread of the OsHV-1 microvariant, which is more similar to the French, Australian, and Chinese microvariants than to the Tomales Bay, California, OsHV-1 variant (Burge et al. 2021). OsHV-1 San Diego Bay microvariant has since been detected during a mass mortality of small juvenile oysters in San Diego Bay in 2020 (Evans et al. submitted). The mitigation of this virus can be successful with selective breeding programs, informed planting times to avoid vulnerable periods based on age, temperature and other environmental factors, appropriate stocking density, and limiting oyster transfer from one location to another (Dégremont et al. 2015, Burge et al. 2017, 2020, Divilov et al. 2019, 2021, Rodgers et al. 2019). Currently, a single oyster nursery exists in San Diego Bay, and the current management strategy is to allow commercial production of oysters only in times when the temperature is <20°C, while sentinel oysters remain for early detection of outbreaks.

In addition to temperature, microbes may also play an important role in OsHV-1 infection. The ability of OsHV-1 to impair host immune defense facilitates opportunistic microbiota to invade and colonize the oyster. Oysters launch a predominantly antibacterial response at later stages of infection, hinting at the likelihood of secondary bacterial infections associated with viral infection (Green et al. 2016). This is further demonstrated by drastic shifts in microbiome community composition and bacterial accumulation with associated tissue damage upon histological examination of infected oysters (de Lorgeril et al. 2018b). A specific group of opportunistic bacteria is conserved across many OsHV-1 infectious environments, including *Arcobacter, Pseudoalteromonas, Amphritea, Marinomonas*, and *Marinobacterium* (de Lorgeril et al. 2018b, Delisle et al. 2022, Pathirana et al. 2022, Clerissi et al. 2023). It is also likely that *Vibrio* species contribute to disease and further its progression after initial infection with OsHV-1 (de Lorgeril et al. 2018b, Petton et al. 2019, Clerissi et al. 2020, 2023, Oyanedel et al. 2023). Some bacteria resulted in co-infections, while others may take advantage of both the host’s immunocompromised state and resources produced by other bacteria, such as siderophores (Oyanedel et al. 2023).

As a result of the limited known detections in San Diego Bay, it is difficult to narrow down an optimal temperature range for the OsHV-1 San Diego Bay microvariant. Therefore, the first goal of this study is to assess the temperature threshold of this microvariant in naïve Pacific oysters without prior exposure to OsHV-1. The second goal was to explore microbial community dynamics after OsHV-1 exposure and assess the impact of bacteria on disease progression. This study has critical implications for OsHV-1 management in San Diego Bay, revealing a tight linkage between temperature and mortality, as well as demonstrating the ability for opportunistic and potentially detrimental bacteria to emerge during viral outbreaks.

## Methods

### Oyster infection trials

Due to the contagious nature of the virus, the experimental room used for infection was an enclosed, climate-controlled room with no flow-through seawater. Waterproof lab boots were worn at all times when in the room, and a 10% bleach bath was prepared for washing the boots after working in the room. Lab coats and boots worn inside the room did not leave the area and were reserved strictly for use in this room. All trash that was accumulated from the experiment was disposed of in double-bagged biohazardous trash bags for off-campus autoclaving. All sharps, including needles and scalpels, were disposed of in biohazardous sharps containers. Drop cloths were placed over tanks to control possible aerosolization of the virus, therefore keeping a barrier between controls and OsHV-1 as a measure of contamination prevention. At the end of the experiment, all containers and materials that were in contact with oysters or OsHV-1 were soaked in 10% bleach for 30 min or sprayed with 10% bleach before rinsing and drying. All protocols were reviewed by the state of California, and the facility was inspected prior to the start of experiments.

#### Oyster infection experiment #1—mortality

To address the first goal of the study, juvenile Pacific oysters were infected with OsHV-1 through bath exposure in experimental aquaria under four different temperatures (15°C, 18°C, 21°C, and 24°C) and monitored mortality for 16 days after exposure.

**Figure 1.**
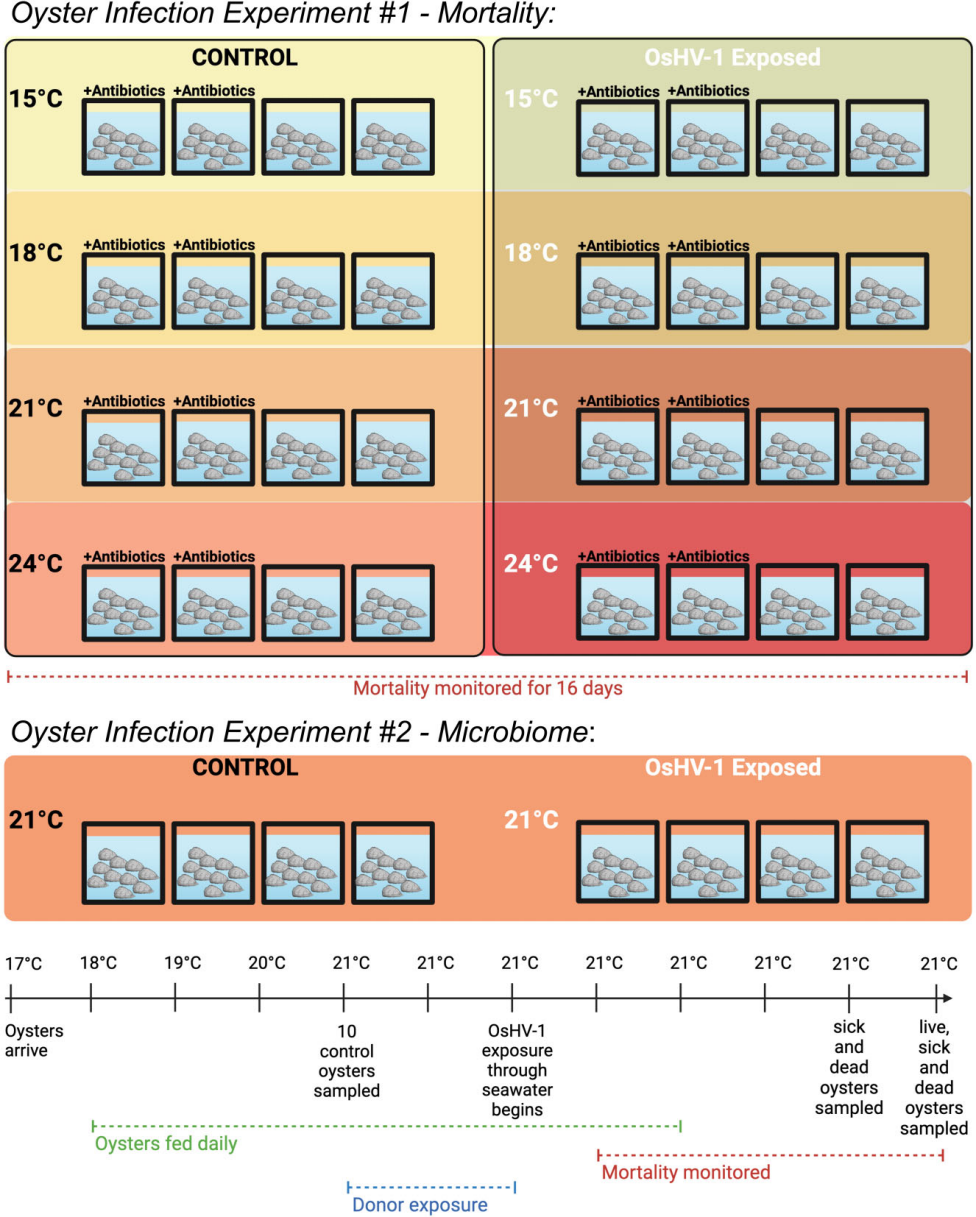
Schematic diagram of the experimental design. Top panel is showcasing mortality experiment setup, while bottom panel is showing microbiome experiment setup. A highly similar timeline as shown in the bottom panel was used for both experiments, with the exception that mortality was monitored for 16 days in the first experiment and oysters were not sampled until they died, while they were all sampled after 4 days in the second experiment. Created with Biorender.com.

Juvenile oysters (either 9 or 24 mm in length) were shipped on ice from an oyster farm in Humboldt Bay, California to Scripps Institution of Oceanography. Oysters had only been exposed to Humboldt Bay water, where OsHV-1 has never been detected (Burge CA, unpublished data, Elston RA unpublished data). All seawater used was from the Scripps Institution of Oceanography pier inlet, was at 35 ppt, and was 0.2 micron-filtered prior to use. Immediately upon arrival, oysters were rinsed briefly with freshwater to clean off sediment and placed into tanks at 15°C or 17°C. Oysters were split into either “donor” or “recipient” tanks. Water infected with OsHV-1 would later be transferred from donor oyster to recipient oyster tanks. The two donor tanks were 39 l Sterilite containers (Sterilite Corporation, USA) with 100 oysters (24 mm length) each starting at 17°C. Recipient tanks were split into two sides: OsHV-1 exposure or Control (Fig. 1). Each side had four water baths to allow for the final acclimation of oysters to four different temperatures (Fig. 1). The recipient tanks were 1 l lidded containers with a hole in the lid for an airline. Ten oysters (9 mm length) were placed in each of these recipient tanks, and four tanks were placed into each water bath (Fig. 1). One water bath on each side (both Control and OsHV-1 exposed) was kept at room temperature, which was 15°C, while all other water baths were started at 17°C by using water heaters. Starting 1 day after the arrival of the oysters, the temperature in the water baths and the donor tanks was increased by 1°C per day until the water reached the desired temperature. Donor tanks were increased to 21°C, while recipient tanks were made to be 15°C 18°C, 21°C, and 24°C (Fig. 1). Temperature loggers were placed in the recipient tank water baths at 21°C to monitor true water temperature fluctuation.

Water in every tank was changed completely every 2 days. All seawater used in the experiment was bleached at 10% before being disposed of down the sink drain. Oysters were fed *ad libitum* every day during the acclimation period. A mix of algae containing 50% *Chaetoceros muelleri*, 5% *Tetraselmis* sp., 20% *Tisochrysis lutea*, and 25% *Nannochloropsis oculata* was boiled at 100°C for 1 min to kill exogenous bacteria before being fed to oysters.

Once all tanks had reached their target water temperatures (21°C for donor oysters or 15°C, 18°C, 21°C, and 24°C for recipient oysters), the donor oysters were placed in a MgCl_2_ bath (50 g/l) overnight to relax their adductor muscle. These donor oysters were injected with 100 µl of OsHV-1 homogenate with a total of 1 × 10^6^ viral copies using a 27-gauge needle injected directly into the adductor muscle. The homogenate used was a secondary pass from the original homogenate (Burge et al. 2021) prepared previously and cryopreserved at −80°C (Kachmar et al. in press). After waiting for 10 min, oysters were placed back into the 39 l containers with filtered seawater at 21°C for 48 h. After 48 h, a water sample was collected from each donor tank, extracted using the Zymo Quick-DNA Miniprep Plus Kit following the Biological Fluids and Cells Protocol, and the concentration of OsHV-1 copies was quantified using qPCR (quantitative polymerase chain reaction) (see full description below). The quantification of virus in the water in each tank was deemed sufficient for exposure (>1 × 10^5^ copies/ml). The water in the two donor tanks was mixed and another sample was taken to determine the final viral concentration in water (4.05 × 10^6^ copies/ml). A volume of 500 ml of the seawater carrying the virus was added to each recipient tank for every temperature on the OsHV-1 exposure side, dosing each set of 10 oysters with 2.03 × 10^9^ copies of OsHV-1. For oysters on the control side, tank water was changed with just filtered seawater.

To assess the magnitude of bacterial impact on OsHV-1 disease, an antibiotic treatment was applied to half of the tanks in all temperatures. For each temperature and condition (OsHV-1 exposed or control), there were four tanks with 10 oysters each (*n* = 32 tanks, *n* = 320 oysters). Two replicate tanks received antibiotics, while the other two replicates did not (Fig. 1). Starting with the first day of viral exposure and continuing at each water change, chloramphenicol was added at a final concentration of 10 mg/l as described in Coffin et al. (2021), Ampicillin at a final concentration of 1 mg/l, and streptomycin at a final concentration of 0.1 mg/ml (Green et al. 2019) were added to the seawater immediately before adding the seawater to the antibiotic-treated tanks. Control tank water did not receive any additives. A follow-up experiment was conducted to validate the lack of difference in mortality between antibiotic and non-antibiotic treated oysters at 18°C. All conditions were replicated, except 20 oysters were used per antibiotic treatment in an OsHV-1 exposure condition, and 10 oysters were used per treatment in the control tanks.

Oysters from all conditions were fed boiled algae immediately after virus exposure and 24 h after virus exposure to ensure filtering of the virus but were starved for the remainder of the experiment. Oysters were monitored daily for mortality. Starting with the control side, tanks were lifted from the water bath, tapped to confirm that live oysters would close, and investigated further if any oysters remained gaping open. Any oysters that remained open were touched lightly with a pair of tweezers. If oysters did not close after this, they were deemed dead and removed from the tank. The tissue of the dead oysters was immediately removed from the shell, weighed, and stored in DNA/RNA shield solution at −20°C (Zymo Research). Once mortality leveled out, with <3 oyster deaths per day for 4 days, the experiment was terminated, and tissue from all remaining live oysters was dissected and preserved in DNA/RNA shield solution.

Oyster tissue was homogenized in the DNA/RNA shield solution for 30 s using the soft tissue tips with the Omni Tissue Homogenizer tool (Omni International). Then, 500 µl of homogenate was used for DNA extraction using the Zymo Quick-DNA Miniprep Plus Kit following the Solid Tissues Protocol. This protocol involves a step of protein digestion for 1 h. The extracted DNA template was stored at −20°C until it was used for quantitative PCR.

#### Oyster infection experiment #2—microbiome

To address the second goal of this study, another bath exposure was performed at 21°C, and oysters were visually assessed and categorized into different disease states (alive, sick, or dead). The microbial patterns in community diversity across disease states and key bacterial players after exposure were compared with other studies to reinforce the theory that a common group of OsHV-1-associated bacteria is conserved across both geographic regions and across different microvariant infections.

Oysters from the nursery in Humboldt Bay were shipped to the climate-controlled room and placed in filtered seawater at 17°C. The temperature of the seawater increased 1°C/day until it reached 21°C. Water was changed every 2 days, and oysters were fed boiled algae. Once oysters reached 21°C, 10 oysters were dissected, weighed, and stored in DNA/RNA shield solution. Then, over 100 donor oysters were relaxed with an MgCl_2_ salt bath overnight and were injected with 100 µl of OsHV-1 homogenate carrying 1 × 10^6^ total copies of OsHV-1 homogenate. After 48 h, a water sample from the tank was collected, extracted, and run through qPCR. The water contained 1.41 × 10^6^ copies/ml of OsHV-1. A volume of 500 ml of this virus water was added to each of six recipient tanks with 10 oysters per tank. After 4 days, 10 dead oysters and two sick oysters were collected from the tanks overall. Dead oysters failed to close after being touched with sterile tweezers, while sick oysters closed slowly and only after the tweezers touched their shells directly. Live oysters closed immediately after tapping the outside of their tank. After 5 days, eight more sick oysters and 10 live oysters were collected from the tanks overall, and the experiment was terminated.

Oyster tissue was homogenized in the DNA/RNA shield solution for 30 s using the soft tissue tips with the Omni Tissue Homogenizer tool and stored at −20°C. Then, 500 µl of homogenate was sent for DNA extraction at the UCSD Microbiome Core using the MagMax Ultra Nucleic Acid Isolation Kit (Applied Biosystems). A portion of extracted DNA template was returned from the Microbiome Core, and viral quantification was performed via qPCR.

### qPCR

Tank water and all oyster tissue samples were run through a qPCR reaction to determine if OsHV-1 was present and at what concentration. Methods were adapted from Agnew et al. (2020) and Burge et al. (2020, 2021). Each qPCR reaction well was prepared with 10 µl of Brilliant III Ultra-Fast SYBR QPCR master mix (Agilent), 0.8 µl of each 10 µM OsHV-1 primer [ORF100 Forward and Reverse (Burge and Friedman 2012, Burge et al. 2021)], 0.5 µl of bovine serum albimun, 5.9 µl of molecular grade sterile H_2_O, and 2 µl of DNA template. All samples were run in triplicate. DNA standards, DNA negative extracts, and qPCR controls were included in every run. The standard curve was prepared using a synthetic gBlock strand of the OsHV-1 ORF100 region (TGATGGATTGTTGGACGAGAGACAACAAGATAGTGCTGAGGATATGAAGAAAAAAGGCCCCAATGTATATAGACCCATGGCCGAAGCGAGAAACATATTTACTTTACAGTCTAATTTTGTAGCGTCCAGGGATGTGAT) in a dilution series from 2 × 10^7^ copies down to 20 copies/µl. Thermocycler (Eppendorf) conditions included one cycle of 20 s at 95°C, followed by 40 cycles of 3 s at 95°C and 30 s at 60°C, and finished with a melt curve profile of 95°C for 15 s, 60°C for 15 s and a ramp up to 95°C held for 15 s. OsHV-1 copy number in unknown oyster and water samples was derived from the standard curve equation for that plate and averaged across the three replicates. Specificity of the amplified template was confirmed by comparing the melt curve of samples to that of the gBlock positive controls.

### Microbiome sequencing

Control (*n* = 10), live (*n* = 10), sick (*n* = 10), and dead (*n* = 10) oyster microbial community libraries were prepared and sequenced by the UCSD Microbiome Core and the Institute for Genomic Medicine at UCSD. Library preparation was conducted using the KAPA Hyper Plus Kit (Roche Diagnostics, USA). The protocol for library preparation and sequencing is based on the Earth Microbiome Project, which uses primers 515F/806R to target the V4 region of the 16S rRNA gene in bacteria (Caporaso et al. 2012, Apprill et al. 2015). Sequencing by synthesis was conducted by the UCSD IGM Genomics Center on the Illumina MiSeq platform with paired-end 250 base pair cycles.

### Analysis

#### Oyster infection experiment #1—mortality

During the 16-day period following OsHV-1 exposure, oysters were monitored every day for mortality. If an oyster was found dead, it was recorded as a “1,” along with its temperature, exposure condition (control versus OsHV-1 Exposed), antibiotic treatment (yes or no), and the days since exposure for when it was found dead. Each mortality was recorded as an individual data point. At the end of the experiment, any remaining live oysters across all variables were recorded individually as “0.” The data on mortality was tracked in this way to perform a survival analysis using the Kaplan–Meier estimate (Kaplan and Meier 1958). In R, the Kaplan–Meier survival estimate was computed with the function *survfit()* from the package *survival* (Therneau and Grambsch 2000, Therneau 2023). Then, the step function plots were created using *ggsurvplot()* from the *survminer* package. Finally, a log rank test was conducted with the *survdiff()* function in the *survival* package to determine whether mortality varied significantly across conditions.

Whole oyster tissue was collected from this experiment, and all tissue was assessed for OsHV-1 viral load. Quantitative PCR data for all OsHV-1 exposed oysters was normalized by weight of tissue in grams, because samples were collected in a standardized volume. The log of the normalized copy numbers by temperature and mortality status (live versus dead) were visualized with violin plots. Kruskall Wallis tests were used to determine whether OsHV-1 copy number varied between live and dead oysters and across temperatures.

#### Oyster infection experiment #2—microbiome

Sequencing reads from the control, alive, sick, and dead oyster samples were demultiplexed with QIIME2 (Bolyen et al. 2019). DADA2 from Qiime2 v2020.6 was used to denoise the data to produce a feature abundance table of Amplicon Sequence Variants (Callahan et al. 2016). Reads were truncated at 224 base pairs on the forward reads and 200 base pairs on the reverse reads based on sequence quality plots. Taxonomic classification of reads was conducted with the Qiime2 pre-trained classifier from Greengenes2 v2022.10 (McDonald et al. 2023). “Unassigned” taxa were filtered out of the feature abundance table. A phylogenetic tree was constructed using the SEPP fragment insertion method (Janssen et al. 2018). The DADA2 ASV abundance table, Greengenes2 taxonomy file, and phylogenetic tree were imported into R as a phyloseq object. Phyloseq v1.42.0 was used to calculate Richness, Evenness, Shannon’s, and Simpson’s alpha diversity (McMurdie and Holmes 2013). Kruskal–Wallis tests with pairwise Dunn’s test were used to determine statistical significance between groups. An alpha correlation test was run to determine strength of linear correlation between evenness and viral load. Richness values were plotted against evenness values as proposed by Gauthier and Derome (2021). A distance matrix was calculated with Unweighted UniFrac using the Phyloseq package and plotted with Principal Coordinates Analysis (Lozupone et al. 2006, McDonald et al. 2018). Adonis tests were run on Unweighted UniFrac distances to determine significance of both disease state and viral load in altering microbial composition. Hierarchical clustering was conducted with the Unweighted UniFrac distance matrix and hclust using the complete linkage method. The tree was cut into its primary five clusters. Differential abundance analysis was conducted with ANCOM-BC (Analysis of Compositions of Microbiomes with Bias Correction), which corrects the bias from uneven sampling fractions across samples, uses a log-linear regression, and conducts multiple pairwise comparisons all while controlling the multi-direction false discovery rate (Lin and Peddada 2020). ANCOM-BC is robust to the sparse matrices used in microbiome data and adaptive to structural versus sampling zeros. The disease state of the oyster sample was used as the fixed formula for ANCOM-BC with an initial prevalence cutoff of 10% and Holm *P*-value adjustment. Pairwise comparisons were conducted against the control samples (before OsHV-1 exposure) as the reference, which produced log fold change values from control to alive, control to sick, and control to dead oyster samples. An adjusted *P*-value of <.05 was used as the cutoff for significantly different taxa, which were agglomerated at the genus level. Finally, Random Forest analysis with *randomForest()* (Liaw and Wiener 2002) package and function in R was performed to assess the ability to predict disease state and OsHV-1 exposure status based on the microbial composition and abundance data. A prevalence cutoff of 10% for each sample set was used again to remove rare taxa. Out of bag error rate was calculated for two comparisons: Alive versus Dead oysters and Control (“Before”) versus Alive & Sick (“After”) oysters. The top 20 ASVs (amplicon sequencing variants) that were most important for predicting condition in either comparison were selected based on the greatest mean decrease in GINI coefficient, which equates to the ASVs improving predictive capabilities at a given node in the decision tree.

## Results

### Oyster infection experiment #1—mortality

**Figure 2.**
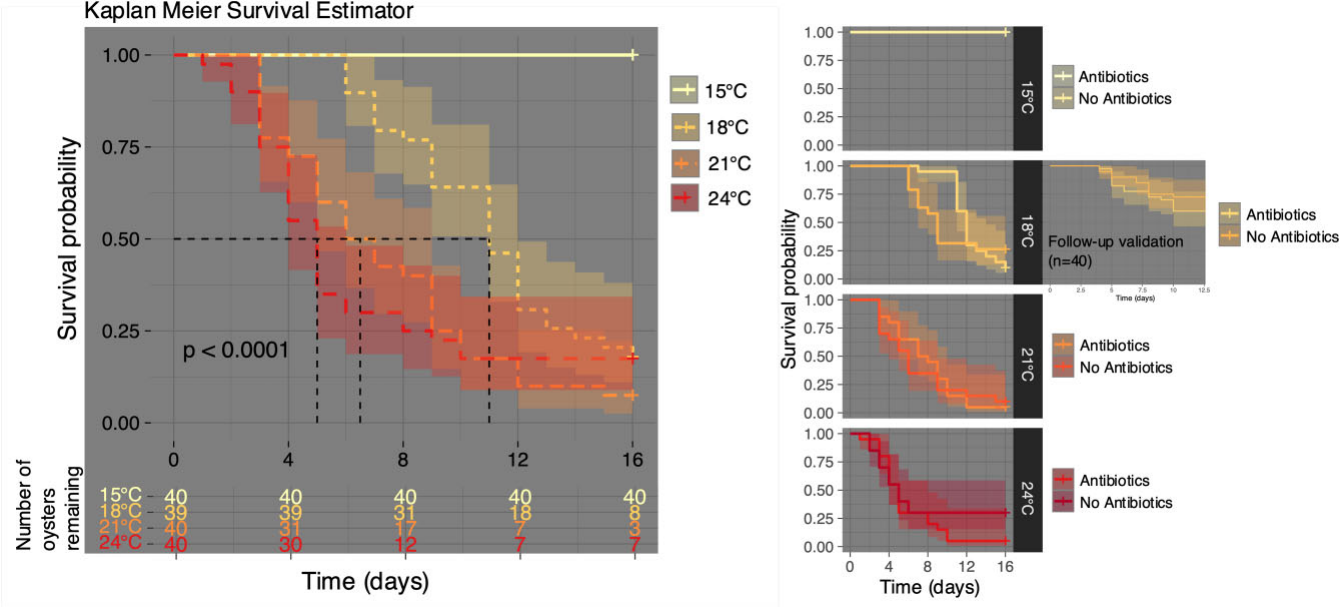
Kaplan–Meier mortality curves displaying the proportion of oysters dead in each condition each day and estimated probability of survival with 95% confidence intervals. Top panel displays survival curves by temperature with an *x*-axis of time in days. Significance of the statistical difference between temperatures is exhibited with *P*-value < .0001. The number of oysters remaining at given time points is displayed below the Kaplan–Meier plot, where the initial sample size of oysters is the first number listed (*n* = 40 for 15°C, 21°C, and 24°C, *n* = 39 for 18°C). Bottom panel displays survival curves by antibiotic exposure, faceted into one plot per temperature. For 18°C, the follow-up validation experiment with 40 oysters is coupled with the original experiment to show trends from the duplicated experiments.

Mortality varied significantly by temperature but did not vary with antibiotic treatment. No oysters in the control group, at any temperature with or without antibiotic treatment died, thus only OsHV-1 exposed oysters were plotted for mortality. At 15°C, no oysters died in any of the temperatures either with or without antibiotic treatment (Fig. 2). In all other temperatures, there was no significant difference in mortality between antibiotic treated or untreated oysters (Fig. 2). Log rank tests were used to determine whether mortality was significantly different between temperatures. Overall, mortality did vary significantly between temperatures (*P* < .0001*) (Fig. 2). Mortality at 18°C was significantly different from that of 15°C (*P* < .0001*), 21°C (*P* = .00065*), and 24°C (*P* = .00227*). However, mortality at 21°C did not vary from 24°C (*P* = .66852). Over 75% of oysters died in all temperatures 18°C and higher, but oysters at 21°C and 24°C started dying sooner after OsHV-1 exposure than oysters at 18°C (Fig. 2). At 18°C, antibiotic treated oysters appear to start dying later than untreated oysters, but this difference was not statistically significant in either the initial or the follow-up experiment (Fig. 2).

**Figure 3.**
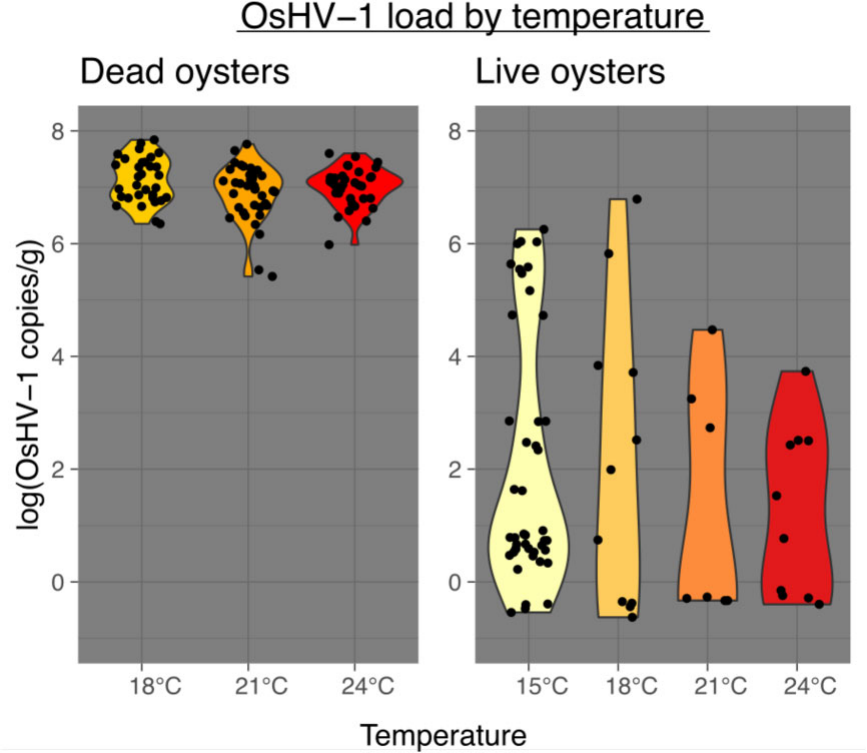
Viral load of OsHV-1 copies is normalized by weight across temperatures faceted by mortality. Copy numbers were determined by qPCR, and each individual dot represents one sample. Violin plots are used to summarize density distribution of values across temperatures. Left panel displays normalized qPCR values for dead oysters sampled throughout the experiment, while the right panel displays values for remaining live oysters collected only at the end of the experiment. 15°C is not included in the left panel as no oysters died at this temperature.

OsHV-1 load was much higher in dead oysters compared to remaining live oysters but did not vary by temperature. Kruskal–Wallis tests were conducted separately for live and dead oysters. Within the dead oysters, OsHV-1 normalized qPCR values did not significantly differ between temperatures (*P* = .168) (Fig. 3). Similarly, OsHV-1 load did not significantly differ between temperatures within remaining live oysters (*P* = .7112) (Fig. 3). A Wilcoxon test (or Mann–Whitney test) was conducted to determine whether OsHV-1 load varied between dead and live oysters across all temperatures combined. OsHV-1 load was significantly higher in dead oysters compared to live oysters (*P* < .001*) (Fig. 3). The variability of normalized qPCR values was much higher in remaining alive oysters, while values for dead oysters were all very similar (Fig. 3).

### Oyster infection experiment #2—microbiome

**Figure 4.**
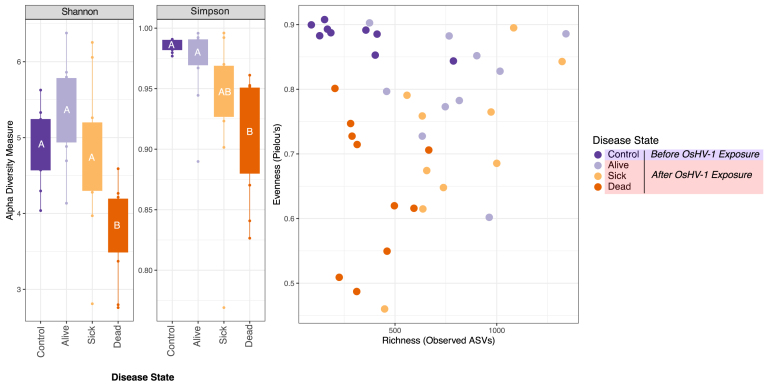
Alpha diversity of oysters before and after exposure to OsHV-1. Left displays Shannon and Simpson diversity with boxplots corresponding to four different disease states: (1) before OsHV-1 exposure, (2) after exposure but still alive, (3) after exposure and showing signs of sickness, and (4) completely dead. Statistically significant difference (*P* < .05) between groups is denoted by “A,” “AB,” and “B,” with “AB” suggesting overlap with both “A” and “B.” Right displays the richness in number of observed ASVs versus the evenness as measured by Pielou’s index as a dot plot for all disease states.

Following infection, the tissue-associated microbial alpha diversity decreased over the spectrum of mortality (alive > sick > dead; Fig. 4). Figure 4 demonstrates two alpha diversity measures across oyster disease states (Shannon’s and Simpson’s) and then breaks down these metrics into their components by plotting Richness against Evenness. Richness and Evenness are both components of Shannon’s and Simpson’s index, with richness having more weight in Shannon’s index and evenness having more weight in Simpson’s index. After exposure to OsHV-1, but while oysters were still alive or sick, Shannon’s and Simpson’s overall diversity measures were unchanged [Kruskal–Wallis (KW) & Dunn’s test (D)/*P* > .05] (Fig. 4). However, Richness increased significantly from before exposure to after (Control—Alive/KW&D/*P* = .0012*; Control—Sick/KW&D/*P* = .0012*) (Fig. 4). Evenness only started to decrease once oysters showed signs of sickness (Control—Sick/KW&D/*P* = .0026*) (Fig. 4). Once oysters were found dead, Shannon’s and Simpson’s diversity decreased significantly (Fig. 4). In dead oysters, Richness returned to a similar level to before OsHV-1 exposure (Control—Dead/KW&D/*P* = .4515) and was significantly lower than remaining sick and alive exposed oysters (Alive—Dead/KW&D/*P* = .0086*; Sick—Dead/KW&D/*P* = .0073*) (Fig. 4). However, Evenness decreased in dead oysters and was significantly lower than oysters before exposure and oysters that were still alive without signs of sickness (Control—Dead/KW&D/*P* = .001*; Alive—Dead/KW&D/*P* = .0228*) (Fig. 4). Evenness was also significantly negatively correlated with viral load. As OsHV-1 load (log(copies/g)) increased, evenness decreased (alpha correlation, *r* = –0.7318, *P* < .0001*).

**Figure 5.**
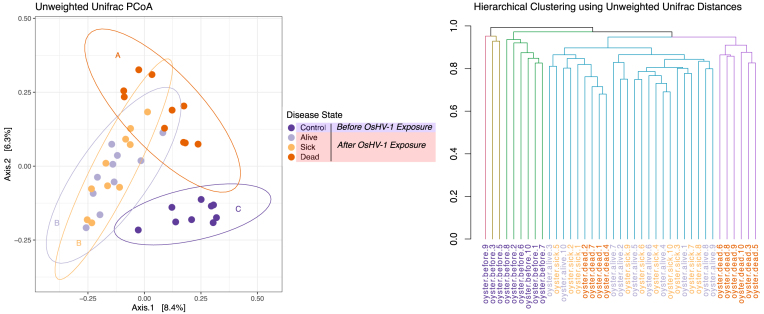
Beta diversity distances between disease states. Left displays a Principal Coordinate Analysis of the Unweighted UniFrac distances between samples. Dots are each a single sample grouped by disease state, and ellipses are added around the groups to demonstrate overlap. Significant differences are denoted by groups “A,” “B,” and “C.” Right displays hierarchical clustering of samples based on the Unweighted UniFrac dissimilarity matrix using the complete linkage method. Sample names are listed along the bottom with reference to their disease state. Samples are split into the five most prevalent clustering groups.

The microbial composition of oyster tissues changes after OsHV-1 exposure and shifts again once the oyster has died. Figure 5 demonstrates the clustering of similar samples based on their microbial communities. Unweighted UniFrac (beta diversity metric) looks at ASVs present but not their abundance. UniFrac also accounts for phylogenetic similarity based on the SEPP fragment insertion phylogenetic tree. Unweighted UniFrac shows significant difference between the microbial constituents of control oysters and alive oysters (adonis, *P* = .006*), as well as control oysters and sick oysters (adonis, *P* = .006*), but not between alive and sick oysters (adonis, *P* = .906) (Fig. 5). Dead oysters also have a significantly different beta diversity compared to control, alive, and sick oysters (adonis, all pairwise comparisons *P* = .006*) (Fig. 5). Unweighted and weighted UniFrac distances are also significantly associated with viral load (adonis, Unweighted: *R*^2^ = 0.064, *P* = .001*, Weighted: *R*^2^ = 0.224, *P* = .001*). Hierarchical clustering breaks the oyster samples into five distinct clusters (Fig. 5). Three clusters are composed of only control oysters, and control oysters are not found in either of the other two clusters (Fig. 5). One cluster contains a mix of alive and sick oysters with a few dead oysters (Fig. 5). The final cluster contains only dead oysters (Fig. 5). The oysters, which cluster into groups together, are most likely to be found in close positions on the Unweighted UniFrac plot as well, suggesting some overlap in the ASVs found in these samples. Most notable is the clear separation between unexposed and OsHV-1-exposed oysters in terms of both Unweighted UniFrac ordination and Hierarchical clustering (Fig. 5).

**Figure 6.**
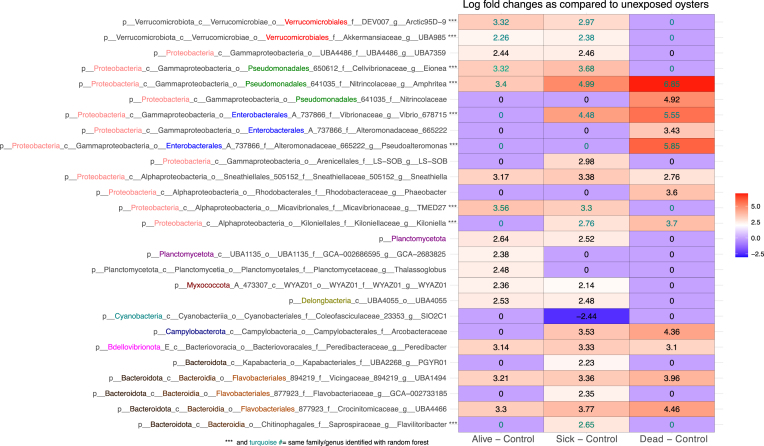
Differential abundance analysis of taxa changing significantly from before to after OsHV-1 exposure. Taxonomic classification down to genus level is provided on the left side of the plot and taxa are organized by phylum. The log fold change of the taxa from (A) control to alive, (B) control to sick, and (C) control to dead is listed in order from left to right. The value of log fold change is written in its respective box for each comparison and taxa, and the box is shaded along a gradient scale from negative to positive values, with a center around 2. Taxa names ending with three asterisks *** indicate that this genus was also identified as one of the top 20 most important in Random Forest analysis for predicting disease state, which are listed in Table 1.

There were 27 genera found to be differentially relatively abundant after conducting pairwise comparisons between control oysters and alive, sick, or dead oysters with ANCOM-BC. The heatmap in Fig. 6 displays significant log fold change values (Holm-adjusted *P*-value < .05) for each genus for each pairwise comparison. Only one genus was found to significantly decrease in abundance from the control group (Fig. 6). The cyanobacteria family Coleofasciculaceae was a significantly lower proportion of the community in the sick oysters (Fig. 6). Many genera were more abundant in both alive and sick oysters compared to control oysters, but not in dead oysters (Fig. 6). These include two genera from Verrucomicrobiales (one of which is from the family Akkermansiaceae), the Proteobacterial groups *Eionea* and Micavibrionaceae, a Planctomycetota, a Myxococcota group, and a Delongbacteria group (Fig. 6). There were also groups that were more abundant in both sick and dead compare to control, but not alive and apparently healthy oysters (Fig. 6). These are *Vibrio, Kiloniella*, and Arcobacteraceae (Fig. 6). No genera were found to be more abundant in both alive and dead, but not sick oysters (Fig. 6). Two Planctomycetota groups (*GCA—2683825* and *Thalassoglobus*) were only more abundant in alive oysters post-OsHV-1 exposure (Fig. 6). Various groups were only more abundant in sick oysters, such as Arenicellales, Kapabacteriales, Flavobacteriaceae, and *Flavilitoribacter* (Fig. 6). Four groups were only more abundant in dead oysters; an unknown genus of Nitrincolaceae, two Alteromonadaceae, one of which was *Pseudoalteromonas*, and *Phaeobacter* (Fig. 6). Finally, various taxa were found to be more abundant in all oyster disease states after OsHV-1 exposure, including *Amphritea* (which increases in log fold change with increasing severity of disease state), *Sneathiella, Peredibacter*, Vicingaceae, and Crocinitomicaceae (Fig. 6).

Of the genera found to be differentially abundant by ANCOM-BC, nine were also identified to be predictive of disease state using Random Forest analysis (Fig. 6, Table 1). These are both Verrucomicrobiales genera, *Eionea, Amphritea, Vibrio, Pseudoalteromonas*, Micavibrionaceae TMED27, *Kiloniella*, and *Flavilitoribacter* (Fig. 6, Table 1). These taxa were most important based on the greatest mean decrease in the Gini coefficient, which means that these taxa increased the ability of the model to predict which group the sample was from. Random Forest models were run on two different subsets of the samples. One model was run on control oysters (*n* = 9) versus alive and sick oysters grouped together (*n* = 20), which was “Before versus After OsHV-1 Infection” (Table 1). Dead oysters were removed from this model because dead oyster tissue likely contains many bacteria involved in decomposition, but not directly associated with OsHV-1 infection. This model had an out of bag error rate of 3.45%. The second model was run on alive (*n* = 10) versus dead (*n* = 10) oysters to determine which bacteria were responsible for moving between disease states once the oyster was already exposed to the virus (Table 1). This model had an out of bag error rate of 10%. Some taxa were found to be important for model prediction in both “Before versus After OsHV-1 infection” and “Alive versus Dead oysters” (Table 1). These include Akkermansiaceae, *Roseovarius*, and *Amphritea spongicola* (Table 1).

**Table 1.** Top 20 most important ASVs for classifying oyster samples in Random Forest analysis.

Top 20 ASV Predictors for Before versus After OsHV-1 Infection	
Predictor Classification	Mean decrease Gini
Bacteroidota|Bacteroidia|Flavobacteriales_877 923|Flavobacteriaceae|*Tenacibaculum_A_805 184*	0.1641
Verrucomicrobiota|Verrucomicrobiae|Verrucomicrobiales|Akkermansiaceae|*Uba985* ***	0.1734
Bacteroidota|Bacteroidia|Chitinophagales|Saprospiraceae|*Lewinella_A*	0.1774
Planctomycetota|B15-G4|B15-G4|B15-G4|*B15-G4*|*Sp003644265*	0.1788
Bacteroidota|Bacteroidia|Flavobacteriales_877 923|Flavobacteriaceae|*Patiriisocius Sp000170815*	0.1891
Proteobacteria|Alphaproteobacteria|Rhodobacterales|Rhodobacteraceae|*Roseovarius_489 432*	0.2040
Campylobacterota|Campylobacteria|Campylobacterales|Arcobacteraceae|*Arcobacter_474 983*	0.2098
Bacteroidota|Bacteroidia|Chitinophagales|Saprospiraceae|*Flavilitoribacter* ***| *Nigricans*	0.2135
Planctomycetota|B15-G4|B15-G4|B15-G4|*B15-G4*| *Sp003644265*	0.2186
Planctomycetota|Planctomycetia|Pirellulales|Pirellulaceae|*Mariniblastus*| *Sp011087765*	0.2268
Bacteroidota|Bacteroidia|Flavobacteriales_877 923|Flavobacteriaceae	0.2348
Proteobacteria|Gammaproteobacteria|Enterobacterales_A_737 866|Vibrionaceae|*Vibrio_678 715* ***	0.2404
Proteobacteria|Alphaproteobacteria|Rhizobiales_A_501 396|Rhizobiaceae_A_499 470	0.2571
Proteobacteria|Alphaproteobacteria|Kiloniellales|Kiloniellaceae|*Kiloniella* ***	0.2871
Proteobacteria|Gammaproteobacteria|Pseudomonadales_641 035|Nitrincolaceae|*Amphritea* ***| *Spongicola*	0.2910
Verrucomicrobiota|Verrucomicrobiae|Verrucomicrobiales|Dev007|*Arctic95d-9* ***	0.2994
Proteobacteria|Alphaproteobacteria|Sphingomonadales|Emcibacteraceae|*Paremcibacter*| *Congregatus*	0.3072
Proteobacteria|Gammaproteobacteria|Pseudomonadales_650 612|Cellvibrionaceae|*Eionea* ***	0.3570
Proteobacteria|Alphaproteobacteria|Micavibrionales|Micavibrionaceae|*Tmed27* ***|*Sp002167715*	0.3641
Proteobacteria|Gammaproteobacteria|Enterobacterales_A_737 866|Kangiellaceae|*Aliikangiella_737 838*| *Coralliicola*	0.4182
**Top 20 ASV Predictors for Alive versus Dead oysters(following OsHV-1 infection)**	
Predictor Classification	Mean decrease gini
Proteobacteria|Gammaproteobacteria|Enterobacterales_A_737 866|Alteromonadaceae_665 222	0.0990
Proteobacteria|Gammaproteobacteria|Enterobacterales_A_737 866|Alteromonadaceae_665 222|P*araglaciecola*	0.1000
Bacteroidota|Bacteroidia|Chitinophagales|Saprospiraceae|*Jaautg01*|*Sp012031785*	0.1145
Bacteroidota|Bacteroidia|Flavobacteriales_877 923|Cryomorphaceae	0.1274
Planctomycetota|Brocadiae|Brocadiales	0.1400
Proteobacteria|Gammaproteobacteria|Enterobacterales_A_737 866|Alteromonadaceae_665 222|Pseudoalteromonas***|*Phenolica*	0.1417
Proteobacteria|Gammaproteobacteria|Enterobacterales_A_737 866|Shewanellaceae_666 538|*Shewanella*| *Colwelliana*	0.1442
Campylobacterota|Campylobacteria|Campylobacterales|Arcobacteraceae	0.1450
Proteobacteria|Alphaproteobacteria|Rhodobacterales|Rhodobacteraceae|*Roseovarius_489 432*	0.1543
Bacteroidota|Bacteroidia|Flavobacteriales_877 923|Flavobacteriaceae	0.1583
Proteobacteria|Alphaproteobacteria|Rhodobacterales|Rhodobacteraceae	0.1720
Calditrichota|Calditrichia|Rbg-13–44-9|J042|*J075*|*Sp003695285*	0.1742
Proteobacteria|Gammaproteobacteria|Enterobacterales_A_737 866|Vibrionaceae|*Vibrio_678 715* ***| *Hanmi*	0.1763
Planctomycetota|Gca-002687715|Gca-002687715|Gca-002687715|*Gca-2683135*| *Sp002683135*	0.1772
Proteobacteria|Gammaproteobacteria|Xanthomonadales_616 050|Marinicellaceae|*Marinicella*| *Sediminis*	0.1829
Proteobacteria|Gammaproteobacteria|Pseudomodales_641 035|Nitrincolaceae|*Amphritea* ***| *Spongicola*	0.1980
Verrucomicrobiota|Verrucomicrobiae|Verrucomicrobiales|Akkermansiaceae|*Uba985* ***	0.1990
Proteobacteria|Gammaproteobacteria|Pseudomonadales_650 612|Pseudohongiellaceae|*Uba9145*	0.2765
Bacteroidota|Bacteroidia|Flavobacteriales_877 923|Flavobacteriaceae|*Maritimimonas*|*Rapae*	0.3192
Proteobacteria|Gammaproteobacteria|Pseudomonadales_650 612|Porticoccaceae|*Porticoccus*	0.4142

ASVs are listed in order of increasing importance. Genus and species are italicized. Red lettering denotes the same ASV in both “Before versus After” and “Alive versus Dead” lists. The three asterisks *** indicate that the genus was identified in both Random Forest and ANCOM-BC analysis.

Taken together, ANCOM-BC and Random Forest results can be more robustly assessed. For example, Akkermansiaceae, *Flavilitoribacter, Vibrio, Kiloniella, Amphritea*, Verrucomicrobiales genus Arctic95d-9, *Eionea*, and Micavibrionaceae TMED27 were all found to be important for predicting whether oysters had been exposed to OsHV-1 or not (Table 1). In ANCOM-BC, these genera were also significantly different between exposed and unexposed oysters, with all groups having increased in the exposed oysters (Fig. 6). In predicting whether oyster samples came from surviving or dead oysters under OsHV-1 exposure, *Pseudoalteromonas, Vibrio, Amphritea*, and Akkermansiaceae were all found to be important (Table 1). In differential abundance analysis, *Pseudoalteromonas, Vibrio*, and *Amphritea* all increased as a proportion of the microbiome in dead oysters (Fig. 6). Akkermansiaceae is significantly increased in surviving oysters, but not in dead oysters (Fig. 6).

## Discussion

### Oyster infection experiment #1—mortality

Mortality in this experiment is expected to be due to the interaction between temperature and OsHV-1. Temperature alone was not responsible for killing oysters, because no control oysters died during experimentation. Marine diseases are tightly linked with temperature. In abalone, Withering Syndrome increases under elevated seawater temperatures (Moore et al. 2000), while abalone herpesvirus infections only occur at temperatures below 23°C (Gu et al. 2019). Both diseases result in mass mortalities. The virus responsible for causing white spot syndrome in shrimp is also highly dependent on temperature, with one study finding that the optimal range for this virus to cause disease is between 23°C and 28°C (Guan et al. 2003). The link between increased water temperatures and disease has been especially well documented in corals, which have been monitored for disease for decades (Harvell et al. 1999, 2002, Burge et al. 2014). Exposure of pathogens to new hosts via range expansion of either the host or the pathogen is in part driven by changes in ocean heat content making new areas suitable for invasion (Harvell et al. 1999, Burge et al. 2014). This may be exacerbated by human-mitigated introduction of new species or populations (Harvell et al. 1999). Spread of Dermo disease and MSX in eastern oysters (*Crassostrea virginica*) on the East Coast of the United States was likely driven by warming ocean temperatures (Harvell et al. 1999, 2002, Burge et al. 2014). Temperature can also impact virulence of pathogens in their current ranges and alter host susceptibility through physiological stress (Harvell et al. 1999, 2002). There may also be overlap in the optimal growth temperature of known pathogens with other opportunistic bacteria, fungi, and viruses, which creates conditions for polymicrobial infections (Harvell et al. 2002).

Colder temperatures have previously been shown to decrease mortality in OsHV-1 exposed oysters (Petton et al. 2013, Pernet et al. 2015, de Kantzow et al. 2016). For the French microvariant, oyster survival is highest at 13°C or lower (Petton et al. 2013, Pernet et al. 2015). For the Australian microvariant, OsHV-1 was not capable of killing oysters when the temperature was dropped to 14°C, and a threshold for productive viral infection and mortality is likely between 14°C and 18°C (de Kantzow et al. 2016). Low temperature likely permits more effective antiviral response by oyster hosts, allowing oysters to survive by limiting viral replication (Green et al. 2014). In the case of the San Diego Bay microvariant assessed in this study, temperatures of 15°C or lower are likely to confer 100% survival against OsHV-1 infection. While quantification of viral copies showed that the virus was able to replicate in at least some oysters at the low temperature, this did not lead to mortality. Therefore, in San Diego Bay, naïve juvenile oysters are unlikely to become lethally infected with OsHV-1 at these low temperatures.

As temperatures increase, the ability of OsHV-1 to infect oysters increases. In this experiment, the optimal temperature for the San Diego Bay OsHV-1 microvariant was 21°C–24°C. Oysters died faster at these temperatures than at 18°C, which may be due to increased metabolism at higher temperatures promoting increased viral replication (Petton et al. 2023). Mortality after just 2 days of bath exposure has been documented previously (Burge et al. 2020). However, overall survival was very low at temperatures above 18°C, which indicates that any temperature that is permissive to the virus creates a big risk for juvenile oysters. In other OsHV-1 variants and microvariants, mortality also increases with temperature (Friedman et al. 2005, Petton et al. 2013, Delisle et al. 2018). The Tomales Bay, CA variant causes outbreaks during the summer that are primarily associated with temperature extremes above 24°C (Friedman et al. 2005, Burge et al. 2006). The French OsHV-1 microvariant tends to be most virulent at temperatures between 16°C and 26°C (Petton et al. 2013, Delisle et al. 2018). Above this temperature range, oyster mortality is decreased, potentially due to altered oyster physiology and more efficient immune response (Petton et al. 2013, Delisle et al. 2018, 2020). The highest temperature tested in this study was 24°C, but it is possible that higher temperatures could inactivate and limit replication of the San Diego Bay OsHV-1 microvariant. For the Australian OsHV-1 microvariant, mortality increases severely at 22°C and 26°C, with 26°C likely being the optimal temperature for inducing mortality (de Kantzow et al. 2016). The San Diego microvariant likely has a similar temperature threshold to other OsHV-1 microvariants, but viral copies are still found in oysters at 15°C after 16 days, suggesting infection is still possible at these lower temperatures if the virus is introduced.

All dead oysters had very similar viral loads regardless of temperature, while remaining alive oysters had highly dispersed viral loads at all temperatures. These dispersed viral loads suggest that individual oysters were at various stages of infection. The viral load and likelihood of mortality also depend highly on genotype, as some families could be more resistant to OsHV-1 infection than others (de Lorgeril et al. 2018b). Burge and Friedman (2012) suggest that viral gene expression is highest in the first few days after infection (Burge and Friedman 2012). Infection may be occurring at different times for each individual oyster, as 10 oysters are housed together, and they may be feeding at different times and rates. Low viral load below 10^6^ copies/mg (Burge et al. 2021) suggests that the oyster was tolerant of the viral infection or was able to become gradually infected from bath exposure and from viral shedding of other oysters throughout the experiment. Higher viral loads in live oysters may be predictive of further mortality, had the oysters been monitored for longer. Oysters may be able to tolerate high viral loads without succumbing to death, which was demonstrated by high viral loads (10^8^ copies) in surviving oysters infected with the French and Australian microvariants (Agnew et al. 2020). Some viral loads in alive oysters overlapped with dead, further suggesting that surpassing a threshold of viral copies may not be the only predictor of death. Although many oysters exposed to OsHV-1 at low temperatures were able to clear the virus, another study showed that OsHV-1 load increased again when moved to warmer temperatures (Pernet et al. 2015). Once the virus is present in an oyster, it is likely latent and can persist until warmer temperatures are able to reactivate it (Petton et al. 2015). This is likely a part of the cause for reactivation during 2020 in San Diego. Outbreaks have only occurred in San Diego Bay in 2018 and 2020, despite temperatures rising well above 18°C every summer. Infected oysters were entirely removed from San Diego following OsHV-1 detection, but OsHV-1 may survive latently in wild oysters or other unknown sources (Burge et al. 2006). OsHV-1 has never been detected in wild oysters in San Diego Bay, but these oysters may be tolerant of the virus and keep viral copies below the limit of detection. Temperature is likely not the only reason for OsHV-1 activation, but additional factors leading to OsHV-1 dispersal are unknown.

Antibiotics had no effect on mortality rates in this experiment. Therefore, the OsHV-1 San Diego Bay microvariant is capable of killing oysters even with a suppressed bacterial community, which differs from other microvariants where *Vibrio* colonization is essential to full disease expression (Petton et al. 2015). As was used in a prior study, chloramphenicol was utilized as an antibiotic to limit bacterial colonization of oysters during OsHV-1 infection (de Lorgeril et al. 2018b). The study by de Lorgeril et al. found that antibiotic treatment significantly reduced mortality in OsHV-1 challenged oysters (de Lorgeril et al. 2018b). Based on gene expression analysis, OsHV-1 infection places oysters in an immunocompromised state, allowing pathogenic bacteria to overcome host immune defenses and leading to lethal bacterial infections (de Lorgeril et al. 2018b). However, the present study did not inoculate *Vibrio* pathogens into the seawater alongside OsHV-1, which would have ensured the presence of a bacterial pathogen with capacity to kill oysters. It is possible that the oysters used in this study did not contain any known pathogenic bacteria from the start of the experiment, which would have been necessary to see the difference between antibiotic treated versus untreated oysters. Alternatively, the viral infection may have been highly potent and efficient in dampening oyster immune response in the specific experimental setting used, whereas a weaker viral infection would have permitted a stronger effect of the antibiotics on survival. Therefore, antibiotics did not offer any additional survival benefit, even if they altered activity of opportunistic bacteria within the oyster microbiome. In other words, the OsHV-1 San Diego Bay microvariant can kill oysters independently of other infections under the right conditions. This experiment focused on juvenile stage oysters, but age has been found to play an important role in OsHV-1 susceptibility (Hick et al. 2018). As such, these results may not directly apply to other age groups, such as oyster seed or adults.

### Oyster infection experiment #2—microbiome

Bacterial community diversity deteriorates as OsHV-1 induced disease progresses to mortality. Microbial alpha diversity metrics, such as Shannon’s and Simpson’s indices, remain unchanged following exposure of Pacific oyster spat to an OsHV-1 microvariant in New Zealand (Delisle et al. 2022), but these metrics do not portray specific changes in either richness or evenness, which can change in themselves without impacting Shannon’s or Simpson’s diversity. Microbe richness increases after OsHV-1 exposure but decreases as the oysters display illness symptoms and then die. Based on previous studies, both absolute abundance of bacteria and number of different bacterial species commonly increase during OsHV-1 infection (de Lorgeril et al. 2018a, 2018b, Clerissi et al. 2023). De Lorgeril et al. (2018a) refer to this period as a destabilization of the bacterial community, which coincides with a decrease in antimicrobial products (de Lorgeril et al. 2018b). Oyster defenses against bacteria are likely suppressed by viral infection, and this disturbance likely promotes the growth of bacteria that were previously susceptible to host immune responses. Once the oyster has died, there is no more host immune response to bacterial invaders and no control over the bacterial community within the tissue. This allows for the bacteria community to shift based on environmental controls and competition for resources rather than host mitigation. At this point, the community has likely shifted to a state of decomposition favoring oyster spoilage bacteria (Chen et al. 2019) and appears to select for dominance of fewer bacterial groups. This is further reflected by the paired drop in evenness when oysters become visibly sick. Bacteria previously promoted by the oyster immune system have likely lost protection with a deficient immune response. As the oysters immune system is weakened by viral proliferation, microbes are also able to proliferate (de Lorgeril et al. 2018a), but competition between bacteria leads to winners and losers in the oyster microbiome. Certain taxa outcompete others and grow in abundance, while many other taxa die out, leaving an uneven distribution of bacterial abundances. In other studies, evenness was found to predict oyster survival against OsHV-1, as resistant oyster families started off with higher bacterial evenness measurements and maintained high evenness during infection (Clerissi et al. 2020). Evenness is an important measure to consider with OsHV-1 infection because the imbalance of bacteria in susceptible oysters is likely an additional stress to the host. Taken together, the changes in richness and evenness are likely due to a weakening immune system brought on by viral infection, which leads to a re-assembling of the bacterial community and a transition toward an opportunist-dominated microbiome and eventually mortality.

Community composition is significantly impacted by OsHV-1 exposure. After OsHV-1 exposure, there is a significant shift toward a new community of bacteria. This community is highly similar between alive and sick oysters (Fig. 5). Multiple studies show a strong shift in the microbiome from before to after OsHV-1 exposure (Clerissi et al. 2020, Delisle et al. 2022), with the greatest change occurring after 24 h of exposure (de Lorgeril et al. 2018b, Clerissi et al. 2023). This change likely occurs alongside exponential viral replication and dysfunction of hemocytes (de Lorgeril et al. 2018b, Clerissi et al. 2023). Although oysters were not sampled until at least 4 days after exposure in this study, the shift in the microbiome likely occurred after just 24 h if the disease behaves similarly to prior studies (de Lorgeril et al. 2018b, Clerissi et al. 2023). There is strong overlap between alive and sick oysters in hierarchical clustering analysis. Some of the dead oysters are also close in microbial composition to alive and sick oysters, one explanation for which is that they died more recently and are yet to decompose. Disease states were subjectively assigned based on behavior and appearance, but microbial composition may do a better job at suggesting disease stage than visual appearance and could even provide an estimation of time since death (Metcalf et al. 2016). Another shift in composition happens once oysters die. Similar to alpha diversity, this likely results from a lack of host control over the microbial community, causing exclusion of certain bacteria and favoring decomposing bacteria. The timing of viral replication is likely the most important factor dictating microbial community composition, as demonstrated in previous studies (de Lorgeril et al. 2018b, Clerissi et al. 2023), as opposed to visual characteristics of exposed oysters. Therefore, the taxa responsible for these shifts in composition may be closely linked to OsHV-1 replication and altered hemocyte function.

Certain bacterial taxa were significantly associated with the transition between disease states. The only bacteria group found to significantly decrease in any comparison was cyanobacterial family Coleofasciculaceae, which was far less represented in sick oysters compared to pre-exposure oysters. This family of cyanobacteria has not been reported in oyster tissue before, preventing a thorough assessment of whether the loss of the bacteria is a sign of declining oyster health. Taxa found in mostly live and sick oysters that were exposed to OsHV-1 may take advantage of the host’s compromised state. In this study, these taxa include Akkermansiaceae, *Flavilitoribacter, Vibrio, Kiloniella, Amphritea*, Verrucomicrobiales genus Arctic95d-9, *Eionea*, and Micavibrionaceae TMED2. Each of these taxa was also important for predicting whether oysters fit into pre- or post-OsHV-1 exposure status. *Vibrio* and *Amphritea* specifically have been identified in association with OsHV-1 infected oysters across many studies (de Lorgeril et al. 2018b, Delisle et al. 2022, Pathirana et al. 2022, Clerissi et al. 2023) and increased in this study with increasing severity of disease state. Additionally, *Arcobacter* was found to be important for distinguishing between exposed and unexposed oyster samples, although the genus itself was not identified in the differential abundance analysis between disease states. Of all these taxa, *Arcobacter, Vibrio*, and *Amphritea* were found to have significantly higher transcriptional activity than other bacteria during OsHV-1 infection in Clerissi et al. (2023). Some of these bacteria may be functionally complementary and take advantage of the weakened host state (Clerissi et al. 2023). On the other hand, certain *Vibrio* species are known to act synergistically with OsHV-1 to accelerate the disease, working in tandem to cause hemocyte damage and impair immune defenses (Oyanedel et al. 2023). *Peredibacter* is another bacteria overrepresented in exposed oysters of all states that has been seen with OsHV-1 infection before but only early on in infection (Clerissi et al. 2023). Other bacteria in this study were found to be primarily associated with mortality or predictive of whether oysters were alive or dead, such as *Pseudoaltermononas, Phaeobacter*, and Cryomorphaceae. *Pseudaltermonoas* and *Phaeobacter* were also found to be associated with OsHV-1 in Clerissi et al. (2023), but only *Pseudaltermonoas* demonstrated high transcriptomic activity during infection (Clerissi et al. 2023). Cryomorphaceae was overrepresented in OsHV-1 infected oysters in both Clerissi et al. (2023) and de Lorgeril et al. (2018b) (de Lorgeril et al. 2018b, Clerissi et al. 2023). In this study, *Pseudalteromonas* and *Phaeobacter* are more unique to dead oyster samples, which may either suggest that they have an important role in the disease progression, alongside *Arcobacter, Vibrio*, and *Amphritea*, or they are just coincidental with dying tissue. *Arcobacter* and *Pseudoalteromonas* were found to be some of predominant spoilage or decomposing bacteria in oyster gills (Chen et al. 2019), but this does not rule out their potential to be opportunistic oyster pathogens. In conclusion, despite differences in OsHV-1 microvariant and temperature thresholds, similar bacteria are found to be associated with OsHV-1 induced disease across many studies. *Arcobacter, Vibrio* and *Amphritea*, and *Pseudoalteromonas* are most likely to be interconnected with OsHV-1 induced disease, and further research should follow-up on their potential to work in concert with OsHV-1 to kill oysters.

## Conclusion

In the present study, mortality of oysters was significantly impacted by the interaction between temperature and OsHV-1 exposure. The San Diego Bay OsHV-1 microvariant can infect, but not kill, oysters when temperatures are 15°C or below. OsHV-1 can kill oysters faster at 21°C and 24°C, which are typical summer temperatures experienced in San Diego Bay. Bacterial communities are significantly altered by OsHV-1 exposure but suppression of bacteria did not significantly lower mortality rates. OsHV-1 may shape bacterial community structure by altering host immune response, leading to an initial increase in bacterial richness followed by a dominance of a few bacteria. The microbiome composition is predictive of disease status, likely more so than visual observation of slow valve closing symptoms. Similar bacteria (*Arcobacter, Vibrio* and *Amphritea*, and *Pseudoalteromonas*) were found to associate with OsHV-1 induced disease across this and multiple other studies despite differences in OsHV-1 microvariants and temperature thresholds. Overall, this study determined that temperature is important for predicting OsHV-1 San Diego Bay microvariant induced mortality, but it is unknown which factors instigate a transmission to previously unexposed oysters in natural conditions in San Diego Bay. This study also importantly recognized a high abundance of four conserved taxa, which are almost always detected in OsHV-1 exposed oysters but had not previously been demonstrated for the San Diego microvariant and oysters from California. Future research should further investigate the interactions between these bacterial taxa and OsHV-1 to better understand their roles in oyster mortality and disease progression.

## Data Availability

All 16S rRNA gene amplicon DNA sequences are deposited publicly in the European Nucleotide Archive at study accession PRJEB72643. The scripts for analyzing data generated in this study are publicly available at: https://github.com/ekunselman/OsHV-1.

## References

[bib1] Abbadi M, Zamperin G, Gastaldelli M et al. Identification of a newly described OsHV-1 µvar from the North Adriatic Sea (Italy). J Gen Virol. 2018;99:693–703. 10.1099/jgv.0.001042 29580370 PMC5994699

[bib2] Agnew MV, Friedman CS, Langdon C et al. Differential mortality and high viral load in naive Pacific Oyster families exposed to OsHV-1 suggests tolerance rather than resistance to infection. Pathogens. 2020;9:1057. 10.3390/pathogens9121057 33348814 PMC7766980

[bib3] Apprill A, McNally S, Parsons R et al. Minor revision to V4 region SSU rRNA 806R gene primer greatly increases detection of SAR11 bacterioplankton. Aquat Microb Ecol. 2015;75:129–37. 10.3354/ame01753

[bib4] Bolyen E, Rideout JR, Dillon MR et al. Reproducible, interactive, scalable and extensible microbiome data science using QIIME 2. Nat Biotechnol. 2019;37:852–7. 10.1038/s41587-019-0209-9 31341288 PMC7015180

[bib5] Burge C, Griffin F, Friedman C. Mortality and herpesvirus infections of the Pacific oyster *Crassostrea gigas* in Tomales Bay, California, USA. Dis Aquat Organ. 2006;72:31–43. 10.3354/dao072031 17067071

[bib6] Burge C, Reece K, Dhar A et al. First comparison of French and Australian OsHV-1 µvars by bath exposure. Dis Aquat Organ. 2020;138:137–44. 10.3354/dao03452 32162612

[bib7] Burge CA, Friedman CS, Kachmar ML et al. The first detection of a novel OsHV-1 microvariant in San Diego, California, USA. J Invertebr Pathol. 2021;184:107636. 10.1016/j.jip.2021.107636 34116033

[bib8] Burge CA, Friedman CS. Quantifying ostreid herpesvirus (OsHV-1) genome copies and expression during transmission. Microb Ecol. 2012;63:596–604. 10.1007/s00248-011-9937-1 21935610

[bib9] Burge CA, Judah LR, Conquest LL et al. Summer seed mortality of the Pacific Oyster, *Crassostrea gigas* Thungberg grown in Tomales Bay, CA, USA: the influence of oyster stock, planting time, pathogens, and environmental stressors. J Shellfish Res. 2007;26:163–72. 10.2983/0730-8000(2007)26[163:SSMOTP]2.0.CO;2

[bib10] Burge CA, Mark Eakin C, Friedman CS et al. Climate change influences on marine infectious diseases: implications for management and society. Ann Rev Mar Sci. 2014;6:249–77. 10.1146/annurev-marine-010213-135029 23808894

[bib11] Burge CA, Shore-Maggio A, Rivlin ND. Ecology of emerging infectious diseases of invertebrates. In: Hajek A.E. (ed.), Ecology of Invertebrate Diseases. Dordrecht: Springer Netherlands, 2017, 587–625.

[bib12] Burioli EAV, Prearo M, Houssin M. Complete genome sequence of ostreid herpesvirus type 1 µVar isolated during mortality events in the Pacific oyster *Crassostrea gigas* in France and Ireland. Virology. 2017;509:239–51. 10.1016/j.virol.2017.06.027 28672223

[bib13] Burioli EAV, Prearo M, Riina MV et al. Ostreid herpesvirus type 1 genomic diversity in wild populations of Pacific oyster *Crassostrea gigas* from Italian coasts. J Invertebr Pathol. 2016;137:71–83. 10.1016/j.jip.2016.05.004 27234424

[bib14] Callahan BJ, McMurdie PJ, Rosen MJ et al. DADA2: high-resolution sample inference from Illumina amplicon data. Nat Methods. 2016;13:581–3. 10.1038/nmeth.3869 27214047 PMC4927377

[bib15] Caporaso JG, Lauber CL, Walters WA et al. Ultra-high-throughput microbial community analysis on the Illumina HiSeq and MiSeq platforms. ISME J. 2012;6:1621–4. 10.1038/ismej.2012.8 22402401 PMC3400413

[bib16] Chen H, Wang M, Yang C et al. Bacterial spoilage profiles in the gills of Pacific oysters (*Crassostrea gigas*) and Eastern oysters (*C. virginica)* during refrigerated storage. Food Microbiol. 2019;82:209–17. 10.1016/j.fm.2019.02.008 31027776

[bib17] Clerissi C, de Lorgeril J, Petton B et al. Microbiota composition and evenness predict survival rate of oysters confronted to Pacific Oyster mortality syndrome. Front Microbiol. 2020;11:311. 10.3389/fmicb.2020.00311 32174904 PMC7056673

[bib18] Clerissi C, Luo X, Lucasson A et al. A core of functional complementary bacteria infects oysters in Pacific Oyster Mortality Syndrome. Animal Microbiome. 2023;5:26. 10.1186/s42523-023-00246-8 37138356 PMC10155333

[bib19] Coffin MRS, Clements JC, Comeau LA et al. The killer within: endogenous bacteria accelerate oyster mortality during sustained anoxia. Limnol Oceanogr. 2021;66:2885–900. 10.1002/lno.11798

[bib20] Davison AJ, Trus BL, Cheng N et al. A novel class of herpesvirus with bivalve hosts. J Gen Virol. 2005;86:41–53. 10.1099/vir.0.80382-0 15604430

[bib21] Dégremont L, Garcia C, Allen SK. Genetic improvement for disease resistance in oysters: a review. J Invertebr Pathol. 2015;131:226–41. 10.1016/j.jip.2015.05.010 26037230

[bib22] de Kantzow M, Hick P, Becker J et al. Effect of water temperature on mortality of Pacific oysters *Crassostrea gigas* associated with microvariant ostreid herpesvirus 1 (OsHV-1 µVar). Aquacult Environ Interact. 2016;8:419–28. 10.3354/aei00186

[bib23] Delisle L, Laroche O, Hilton Z et al. 2022. Understanding the dynamic of POMS infection and the role of microbiota composition in the survival of Pacific oysters, *Crassostrea gigas*. https://www.researchsquare.com (16 Jun 2022, date last accessed). 10.1128/spectrum.01959-22 PMC9769987 36314927

[bib24] Delisle L, Pauletto M, Vidal-Dupiol J et al. High temperature induces transcriptomic changes in *Crassostrea gigas* that hinders progress of ostreid herpesvirus (OsHV-1) and promotes survival. J Exp Biol. 2020;223: jeb226233. 10.1242/jeb.226233 32816959 PMC7578350

[bib25] Delisle L, Petton B, Burguin JF et al. Temperature modulate disease susceptibility of the Pacific oyster *Crassostrea gigas* and virulence of the ostreid herpesvirus type 1. Fish Shellfish Immunol. 2018;80:71–79. 29859311 10.1016/j.fsi.2018.05.056

[bib26] de Lorgeril J, Escoubas J-M, Loubiere V et al. Inefficient immune response is associated with microbial permissiveness in juvenile oysters affected by mass mortalities on field. Fish Shellfish Immunol. 2018a;77:156–63. 29567138 10.1016/j.fsi.2018.03.027

[bib27] de Lorgeril J, Lucasson A, Petton B et al. Immune-suppression by OsHV-1 viral infection causes fatal bacteraemia in Pacific oysters. Nat Commun. 2018b;9:4215. 10.1038/s41467-018-06659-3 30310074 PMC6182001

[bib28] Divilov K, Schoolfield B, Mancilla Cortez D et al. Genetic improvement of survival in Pacific oysters to the Tomales Bay strain of OsHV-1 over two cycles of selection. Aquaculture. 2021;543:737020. 10.1016/j.aquaculture.2021.737020

[bib29] Divilov K, Schoolfield B, Morga B et al. First evaluation of resistance to both a California OsHV-1 variant and a French OsHV-1 microvariant in Pacific oysters. BMC Genet. 2019;20:96. 10.1186/s12863-019-0791-3 31830898 PMC6909534

[bib30] Friedman C, Estes R, Stokes N et al. Herpes virus in juvenile Pacific oysters *Crassostrea gigas* from Tomales Bay, California, coincides with summer mortality episodes. Dis Aquat Organ. 2005;63:33–41. 10.3354/dao063033 15759798

[bib31] Gauthier J, Derome N. Evenness-richness scatter plots: a visual and insightful representation of shannon entropy measurements for ecological community analysis. mSphere. 2021;6:e01019–20. 10.1128/mSphere.01019-20 33827912 PMC8546709

[bib32] Green TJ, Montagnani C, Benkendorff K et al. Ontogeny and water temperature influences the antiviral response of the Pacific oyster, *Crassostrea gigas*. Fish Shellfish Immunol. 2014;36:151–7. 24200990 10.1016/j.fsi.2013.10.026

[bib33] Green TJ, Siboni N, King WL et al. Simulated marine heat wave alters abundance and structure of Vibrio populations associated with the Pacific oyster resulting in a mass mortality event. Microb Ecol. 2019;77:736–47. 10.1007/s00248-018-1242-9 30097682

[bib34] Green TJ, Vergnes A, Montagnani C et al. Distinct immune responses of juvenile and adult oysters (*Crassostrea gigas*) to viral and bacterial infections. Vet Res. 2016;47:72. 10.1186/s13567-016-0356-7 27439510 PMC4955271

[bib35] Gu L, Qi R-J, Yang R et al. The prevalence of abalone herpesvirus in two Haliotis species in South China during 2002–2013. Aquaculture. 2019;505:18–26. 10.1016/j.aquaculture.2019.02.026

[bib36] Guan Y, Yu Z, Li C. The effects of temperature on white spot syndrome infections in *Marsupenaeus japonicus*. J Invertebr Pathol. 2003;83:257–60. 10.1016/S0022-2011(03)00068-5 12877834

[bib37] Harvell CD, Kim K, Burkholder JM et al. Emerging marine diseases—climate links and anthropogenic factors. Science. 1999;285:1505–10. 10.1126/science.285.5433.1505 10498537

[bib38] Harvell CD, Mitchell CE, Ward JR et al. Climate warming and disease risks for terrestrial and marine biota. Science. 2002;296:2158–62. 10.1126/science.1063699 12077394

[bib39] Hick PM, Evans O, Rubio A et al. Both age and size influence susceptibility of Pacific oysters (*Crassostrea gigas*) to disease caused by ostreid herpesvirus -1 (OsHV-1) in replicated field and laboratory experiments. Aquaculture. 2018;489:110–20. 10.1016/j.aquaculture.2018.02.013

[bib40] Janssen S, McDonald D, Gonzalez A et al. Phylogenetic placement of exact amplicon sequences improves associations with clinical information. Msystems. 2018;3:e00021–18. 10.1128/mSystems.00021-18 29719869 PMC5904434

[bib41] Jenkins C, Hick P, Gabor M et al. Identification and characterisation of an ostreid herpesvirus-1 microvariant (OsHV-1 µ-var) in *Crassostrea gigas* (Pacific oysters) in Australia. Dis Aquat Organ. 2013;105:109–26. 10.3354/dao02623 23872855

[bib42] Kaplan EL, Meier P. Nonparametric estimation from incomplete observations. J Am Statist Assoc. 1958;53:26. 10.1080/01621459.1958.10501452

[bib43] Keeling S, Brosnahan C, Williams R et al. New Zealand juvenile oyster mortality associated with ostreid herpesvirus 1—an opportunistic longitudinal study. Dis Aquat Organ. 2014;109:231–9. 10.3354/dao02735 24991849

[bib44] Liaw A, Wiener M. Classification and regression by randomForest. R News. 2002;2:18–22.

[bib45] Lin H, Peddada SD. Analysis of compositions of microbiomes with bias correction. Nat Commun. 2020;11:3514. 10.1038/s41467-020-17041-7 32665548 PMC7360769

[bib46] Lozupone C, Hamady M, Knight R. UniFrac—an online tool for comparing microbial community diversity in a phylogenetic context. BMC Bioinf. 2006;7:371. 10.1186/1471-2105-7-371 PMC1564154 16893466

[bib47] Lynch SA, Carlsson J, Reilly AO et al. A previously undescribed ostreid herpes virus 1 (OsHV-1) genotype detected in the pacific oyster, *Crassostrea gigas*, in Ireland. Parasitology. 2012;139:1526–32. 10.1017/S0031182012000881 23036593

[bib48] Martenot C, Faury N, Morga B et al. Exploring first interactions between ostreid herpesvirus 1 (OsHV-1) and its host, *Crassostrea gigas*: effects of specific antiviral antibodies and dextran sulfate. Front Microbiol. 2019;10:1128. 10.3389/fmicb.2019.01128 31178841 PMC6543491

[bib49] Martenot C, Fourour S, Oden E et al. Detection of the OsHV-1 μVar in the Pacific oyster *Crassostrea gigas* before 2008 in France and description of two new microvariants of the ostreid herpesvirus 1 (OsHV-1). Aquaculture. 2012;338–341:293–6. 10.1016/j.aquaculture.2011.12.030

[bib50] Martenot C, Gervais O, Chollet B et al. Haemocytes collected from experimentally infected Pacific oysters, *Crassostrea gigas*: detection of ostreid herpesvirus 1 DNA, RNA, and proteins in relation with inhibition of apoptosis. PLoS One. 2017;12:e0177448. 10.1371/journal.pone.0177448 28542284 PMC5436676

[bib51] Martenot C, Oden E, Travaillé E et al. Comparison of two real-time PCR methods for detection of ostreid herpesvirus 1 in the Pacific oyster *Crassostrea gigas*. J Virol Methods. 2010;170:86–89. 10.1016/j.jviromet.2010.09.003 20837066

[bib52] Mazaleyrat A, Normand J, Dubroca L et al. A 26-year time series of mortality and growth of the Pacific oyster C. gigas recorded along French coasts. Sci Data. 2022;9:392. 10.1038/s41597-022-01511-2 35810155 PMC9271045

[bib53] McDonald D, Jiang Y, Balaban M et al. Greengenes2 unifies microbial data in a single reference tree. Nat Biotechnol. 2023;42:715–718. 37500913 10.1038/s41587-023-01845-1 PMC10818020

[bib54] McDonald D, Vázquez-Baeza Y, Koslicki D et al. Striped UniFrac: enabling microbiome analysis at unprecedented scale. Nat Methods. 2018;15:847–8. 10.1038/s41592-018-0187-8 30377368 PMC7250580

[bib55] McMurdie PJ, Holmes S. phyloseq: an R Package for reproducible interactive analysis and graphics of microbiome census data. PLoS One. 2013;8:e61217. 10.1371/journal.pone.0061217 23630581 PMC3632530

[bib56] Metcalf JL, Xu ZZ, Weiss S et al. Microbial community assembly and metabolic function during mammalian corpse decomposition. Science. 2016;351:158–62. 10.1126/science.aad2646 26657285

[bib57] Moore JD, Robbins TT, Friedman CS. Withering syndrome in farmed red abalone haliotis rufescens: thermal induction and association with a gastrointestinal rickettsiales-like prokaryote. J Aquat Anim Health. 2000;12:26–34. 10.1577/1548-8667(2000)012<0026:WSIFRA>2.0.CO;2 28880772

[bib58] Oyanedel D, Lagorce A, Bruto M et al. Cooperation and cheating orchestrate Vibrio assemblages and polymicrobial synergy in oysters infected with OsHV-1 virus. Proc Natl Acad Sci. 2023;120:e2305195120. 10.1073/pnas.2305195120 37751557 PMC10556616

[bib59] Pathirana E, Whittington RJ, Hick PM. Impact of seawater temperature on the Pacific oyster (*Crassostrea gigas*) microbiome and susceptibility to disease associated with ostreid herpesvirus-1 (OsHV-1). Anim Prod Sci. 2022;62:1040–54. 10.1071/AN21505

[bib60] Peeler EJ, Allan Reese R, Cheslett DL et al. Investigation of mortality in Pacific oysters associated with ostreid herpesvirus-1μVar in the Republic of Ireland in 2009. Prev Vet Med. 2012;105:136–43. 10.1016/j.prevetmed.2012.02.001 22398251

[bib61] Pernet F, Tamayo D, Petton B. Influence of low temperatures on the survival of the Pacific oyster (*Crassostrea gigas*) infected with Ostreid herpes virus type 1. Aquaculture. 2015;445:57–62. 10.1016/j.aquaculture.2015.04.010

[bib62] Petton B, Alunno-Bruscia M, Mitta G et al. Increased growth metabolism promotes viral infection in a susceptible oyster population. Aquacult Env Interact. 2023;15:19–33. 10.3354/aei00450

[bib63] Petton B, Bruto M, James A et al. *Crassostrea gigas* mortality in France: the usual suspect, a herpes virus, may not be the killer in this polymicrobial opportunistic disease. Front Microbiol. 2015;6:686. 10.3389/fmicb.2015.00686 26217318 PMC4491618

[bib64] Petton B, de Lorgeril J, Mitta G et al. Fine-scale temporal dynamics of herpes virus and vibrios in seawater during a polymicrobial infection in the Pacific oyster *Crassostrea gigas*. Dis Aquat Organ. 2019;135:97–106. 10.3354/dao03384 31342911

[bib65] Petton B, Pernet F, Robert R et al. Temperature influence on pathogen transmission and subsequent mortalities in juvenile Pacific oysters *Crassostrea gigas*. Aquacult Env Interact. 2013;3:257–73. 10.3354/aei00070

[bib66] Picot S, Faury N, Pelletier C et al. Monitoring autophagy at cellular and molecular level in *Crassostrea gigas* during an experimental ostreid herpesvirus 1 (OsHV-1) infection. Front Cell Infect Microbiol. 2022;12:858311. 10.3389/fcimb.2022.858311 35444958 PMC9014014

[bib67] Rodgers C, Arzul I, Carrasco N et al. A literature review as an aid to identify strategies for mitigating ostreid herpesvirus 1 in *Crassostrea gigas* hatchery and nursery systems. Rev Aquacult. 2019;11:565–85. 10.1111/raq.12246

[bib68] Schikorski D, Renault T, Saulnier D et al. Experimental infection of Pacific oyster *Crassostrea gigas* spat by ostreid herpesvirus 1: demonstration of oyster spat susceptibility. Vet Res. 2011;42:27. 10.1186/1297-9716-42-27 21314910 PMC3042938

[bib69] Segarra A, Pépin JF, Arzul I et al. Detection and description of a particular ostreid herpesvirus 1 genotype associated with massive mortality outbreaks of Pacific oysters, *Crassostrea gigas*, in France in 2008. Virus Res. 2010;153:92–99. 10.1016/j.virusres.2010.07.011 20638433

[bib70] Therneau TM, Grambsch PM. Modeling Survival Data: Extending the Cox Model. New York: Springer, 2000.

[bib71] Therneau TM. A Package for survival analysis in R, 2023. https://CRAN.R-project.org/package=survival

[bib72] Wiltshire KH. Ecophysiological Tolerances of the Pacific oyster, Crassostreagigas, with Regard to the Potential Spread of Populations in South Australian waters. South Australian Research and Development Institute (Aquatic Sciences), Prepared for PIRSA Marine Biosecurity No. F2007/000499-1, 2007.

